# A new method for treating fecal incontinence by implanting stem cells derived from human adipose tissue: preliminary findings of a randomized double-blind clinical trial

**DOI:** 10.1186/s13287-017-0489-2

**Published:** 2017-02-21

**Authors:** Arash Sarveazad, Graham L. Newstead, Rezvan Mirzaei, Mohammad Taghi Joghataei, Mehrdad Bakhtiari, Asrin Babahajian, Bahar Mahjoubi

**Affiliations:** 1 Colorectal Research Center, Iran University of Medical Sciences, Tehran, Iran; 2 Sydney Colorectal Associates, Prince of Wales Hospital, Randswick, NSW Australia; 3 Cellular and Molecular Research Center, Faculty of Medicine, Iran University of Medical Sciences, Tehran, Iran; 4 Liver and Digestive Research Center, Kurdistan University of Medical Sciences, Sanandaj, Iran

**Keywords:** Fecal incontinence, Stem cells derived from human adipose tissue, Human, Clinical trial

## Abstract

**Background:**

Anal sphincter defects are a major cause of fecal incontinence causing negative effects on daily life, social interactions, and mental health. Because human adipose-derived stromal/stem cells (hADSCs) are easier and safer to access, secrete high levels of growth factor, and have the potential to differentiate into muscle cells, we investigated the ability of hADSCs to improve anal sphincter incontinence.

**Methods:**

The present randomized double-blind clinical trial was performed on patients with sphincter defects. They were categorized into a cell group (*n* = 9) and a control group (*n* = 9). Either 6 × 10^6^ hADSCs per 3 ml suspended in phosphate buffer saline (treatment) or 3 ml phosphate buffer saline (placebo) was injected. Two months after surgery, the Wexner score, endorectal sonography, and electromyography (EMG) results were recorded.

**Results:**

Comparing Wexner scores in the cell group and the control group showed no significant difference. In our EMG and endorectal sonography analysis using ImageJ/Fiji 1.46 software, the ratio of the area occupied by the muscle to total area of the lesion showed a 7.91% increase in the cell group compared with the control group.

**Conclusion:**

The results of the current study show that injection of hADSCs during repair surgery for fecal incontinence may cause replacement of fibrous tissue, which acts as a mechanical support to muscle tissue with contractile function. This is a key point in treatment of fecal incontinence especially in the long term and may be a major step forward.

**Trial registration:**

Iranian Registry of Clinical Trials IRCT2016022826316N2. Retrospectively registered 7 May 2016.

## Background

Pelvic floor disorders caused by weakness of pelvic muscles, connective tissue, and fascia certainly can affect quality of life and lead to increased healthcare costs [1]. One of the major pelvic floor disorders is due to damage to, or a defect in, the anal sphincter, which is in itself a major cause of fecal incontinence; such incontinence is primarily due to reduction in mechanical pressure in the anal sphincter and thus the inability to close the anal canal [2, 3]. It is clear that fecal incontinence has negative effects on daily life, social interactions, and mental health [4, 5]. Although fecal incontinence affects people of all age groups, it is particularly common in women (mostly due to injury during labor) and also in older people [6]. The prevalence of fecal incontinence has been reported at 6% in women younger than 40 years old, 15% in women older than 40 years and 6–10% in men [7]. Although surgical repair of the anal sphincter is the main treatment approach for treating fecal incontinence caused by anatomical defects [8], the overall results of this approach are not satisfactory and recurrence of incontinence, especially in long-term follow-up, is common [9–12]. Moreover, alternative methods such as artificial sphincters are not ideal due to the risks of device failure [13]. The effects of other methods such as injection of bulking agents may be limited by absorption or migration of the injected agent, fat embolism, or granuloma formation [14, 15]. Thus, regeneration of the lost anal sphincter muscle tissue and improvement of its function using stem cells might be considered as an alternative treatment strategy. Easier and safer access [16–18], production of multiple growth factors [19–21], and secretion of high levels of angiogenic factors [22] in human adipose-derived stromal/stem cells (hADSCs) makes them an appropriate option for muscle repair. The feasibility and safety of hADSCs have been approved in some phase I clinical trials [23, 24]. The ability of hADSCs to differentiate into muscle cells has been clearly shown in both in-vitro [25–28] and in-vivo [29–33] studies. This study aims to answer the question of whether stem cell therapy or hADSCs can improve anal sphincter lesions after sphincteroplasty. Following anal sphincter repair, because the repair site will be replaced with fibrous tissue, long-term function of the sphincter has not been better than its short-term function. Thus, the present study aims to determine the long-term consequences of hADSC implantation and compare these with short-term results. The results of this study were initially assessed at 2 months, while the patients are now under observation to determine their long-term results.

## Methods

### Study design and setting

The present randomized double-blind clinical trial was carried out in 2012 and 2013 to evaluate the efficacy of allogeneic transplantation of hADSCs for relieving fecal incontinence in patients with external anal sphincter defects presenting to Hazrate Rasoole Akram Hospital, Tehran, Iran. In this study, patients were consecutively entered and divided into two equal groups of sphincteroplasty with hADSC transplant (cell group) and sphincteroplasty with phosphate buffer saline (PBS; control group). The protocol of the study was approved by the Ethics Committee of Iran University of Medical Sciences. Researchers adhered to the principles of the Helsinki Declaration throughout the study and written informed consent was obtained from the patients. The protocol of the study was registered in the Iranian Registry of Clinical Trials (IRCT2016022826316N2).

### Selection of patients

**Fig. 1:**
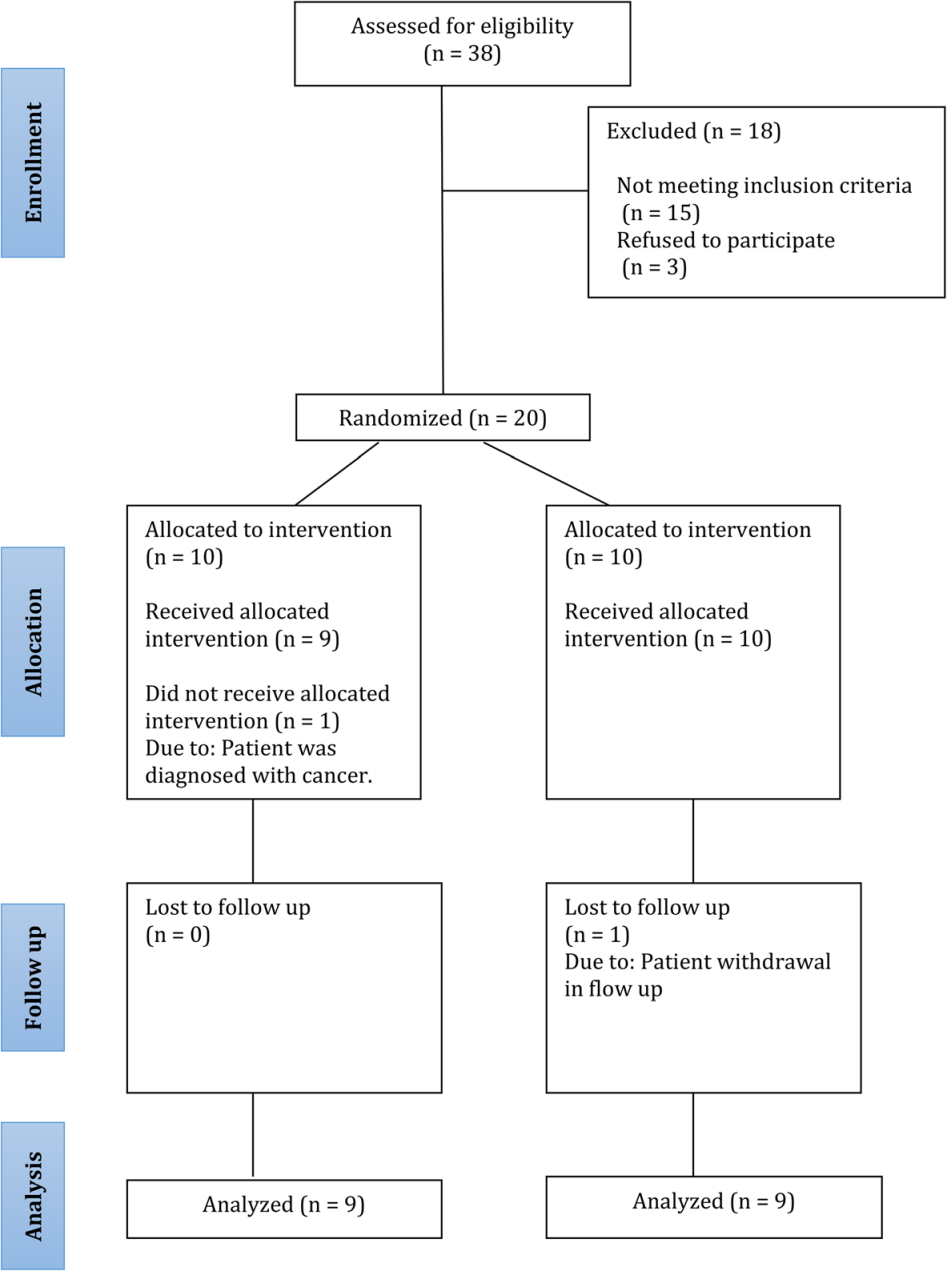
CONSORT flowchart of the present study

The present study was performed on 18 patients (15 women and 3 men, aged 25–78 years) who were randomly assigned to either the cell group (*n* = 9) or the control group (*n* = 9) (Fig. 1). Eligibility criteria are presented in Table 1. After taking their history and confirming fecal incontinence based on the Wexner score, [34] all subjects underwent 2D and 3D endorectal ultrasonography with a 360° probe (BK Pro Focus type 2202; OR, USA; Supplier: SafirMed) to assess sphincter damage.

**Table 1:** Eligibility criteria of patients

Inclusion criteria	Exclusion criteria
− Age > 18 years − Wexner score ≥ 8 − Confirmation of external anal sphincter defect with endorectal ultrasonography − Patient consent for inclusion	− Pregnancy and breastfeeding − Participating in other trials within the past 30 days − History of artificial sphincter − Vaginal delivery within the past 6 months − Presence of chronic diseases − Allergy to bovine-derived materials − Autoimmune disease

A permuted block randomization (block size 3) was used to implement the random allocation sequence without stratification for baseline characteristics. An independent physician generated the random allocation sequence by a computer-based program, enrolled participants, and assigned participants to interventions. Four separate researchers prepared the suspensions, carried out the treatment administration, and recorded patients’ data. The patients and the surgeons who performed the surgery, sonographic, and physical examinations were blinded to the type of treatment. To ensure the blinding of the surgeon to the type of treatment, the cell suspension (6 × 10^6^ cells per 3 ml PBS) and placebo (3 ml PBS) were both prepared in packages which appeared to be the same.

### Cell preparation

The isolation, preparation, and characterization of hADSCs, as well as corresponding results, were presented previously [35].

### Isolation of hADSCs

Fat tissue was prepared from the superficial abdominal fat tissue layer in individuals aged 25–35 years who were referred to the general ward of Rasoul-e-Akram Hospital in Tehran, Iran for liposuction surgery after obtaining informed consent. The eligible patients had no history of positive HIV or hepatitis infections. Sampling was done in sterile conditions. The samples were transferred to the laboratory in a sterile plate containing DMEM/Hams F-12, FBS 10%, and penicillin/streptomycin (P/S) 5%. Isolation of stem cells was carried out based on a protocol described by Dubois et al. [36]. The fat samples were heated to 37 °C in a water bath prior to extraction. All of the extraction procedures were carried out under a sterile hood with sterile equipment. Washing the adipose tissue was performed in a tube containing P/S 1% solution (prepared by warming the PBS) and blood vessels and connective tissue were harvested to clear the tissue. The sample was transferred to a tube containing collagenase 0.1% and BSA 1.0% for tissue digestion. The tube containing the sample was then stored in a water bath for 30 minutes to complete digestion of the tissue samples. After tissue digestion, the tube containing the sample was centrifuged at a speed of 1200 rpm at room temperature for 5 minutes. After draining the supernatant fluid, the formed pellet was resuspended in a solution of BSA 1%, and centrifugation was carried out once again. To remove red blood cells, the formed pellet was resuspended with RBC lysis buffer and centrifuged. Finally, after washing with PBS, centrifugation, and evacuation of supernatant fluid, the reformed pellet was resuspended in DMEM/Hams F-12 culture medium containing FBS 10% and P/S 1% and was then transferred to a flask. Flasks were kept in the incubator (37 °C, CO_2_ 5%, moisture 98%) until the fifth passage.

### hADSC characterization

Flow cytometry was used to identify and confirm the hADSCs. After medium evacuation and washing with PBS, cells in flasks ready for the fifth passage were isolated from the flask by proximity to trypsin 25% for 4 minutes in the incubator. The cells were then used for flow cytometry after enzymatic neutralization, which was achieved by adding the same volume of medium and then transferring to a test tube. The cells were incubated with anti-CD45, anti-CD44 (conjugated to FITC), anti-CD90, anti-CD73, or anti-CD31 (conjugated to PE) for 30 minutes. All antibodies were purchased from BD Biosciences.

### Surgical procedure

For the patients suffering from fecal incontinence due to sphincter damage, sphincteroplasty was performed. In this procedure, after opening the wound and excising the scar, the two ends of the muscle were exposed and washed with normal saline. The ends were then re-approximated without tension; 3-0 PDS sutures were utilized for repair. After washing with normal saline profusely and controlling any bleeding, 6 × 10^6^ hADSCs (3 × 10^6^ per 1.5 ml PBS into each end of the muscle) were injected slowly. The repair was then covered internally by an endorectal flap. Finally, outside the sphincter, excess skin tags were removed. The surgical procedure was completed without injection of antibiotics and antiseptics. Wound dressing was performed with gauze soaked in saline. In the following days, the wound was washed with abundant saline and fresh dressings applied. In the control group, all procedures were performed in the same manner but without implanting hADSCs.

In cases with external anal sphincter damage-related high trans-sphincteric fistula, it was usually impossible and inappropriate to primarily repair the sphincter because of the presence of extensions and collections around and above the fistula after laying them open. These wounds were left open and hADSCs (3 × 10^6^ per 1.5 ml in each end of the muscle) were slowly injected a few millimeters from the edge of the muscle.

### Safety assessment

The present study evaluated the safety of hADSCs, which consisted of assessing the reported side effects after surgery until discharge from hospital and a 2-month follow-up of patients. Every 2 weeks, a full history was taken from the patients, and at the end of the second month, blood, urine, and stool analyses were performed. Safety of hADSCs was evaluated based on the WHO toxicity scale, in which the severity of adverse events is divided into four grades, namely mild, moderate, severe, and life-threatening [37]. Based on this scale, hematologic, chemistry, pancreatic and liver enzyme, stool, and urine analyses as well as cardiovascular, respiratory, digestive, neurologic, skin, and systemic (allergic reaction, headache, fever, and malaise) evaluations were done for all patients. Finally, mortality of the patients was also assessed.

### Outcomes

The initial plan was to undertake a biopsy before final wound closure (after 8 weeks) and histopathological assessment of the repair zone for differentiation of muscle and fibrous tissue. However, due to the early closure of many wounds, obtaining a biopsy for histologic examination was not possible. Therefore, to check the results and the differentiation of fibrous tissue from muscle, sonography and electromyography (EMG) were used instead of histopathological examination.

### Wexner score

To check muscle function after complete healing, the Wexner score was recorded by a single colorectal surgeon blinded to the study protocol.

### Endorectal sonography

Two months after surgery, 2D and 3D trans-anal endosonography were performed to assess the integrity of the anal sphincter muscle as well as to detect muscle in the gap created in the repair site. Three sonographic images of the lesion site were then taken and the amount of muscle was determined using ImageJ/Fiji 1.46 software and the following equation:

$$ \mathrm{Percent}\kern0.5em \mathrm{of}\kern0.5em \mathrm{muscle}\kern0.5em \mathrm{occupied}\kern0.5em \mathrm{area}\kern0.5em =\kern0.5em \frac{\mathrm{Area}\kern0.5em \mathrm{occupied}\kern0.5em \mathrm{by}\kern0.5em \mathrm{the}\kern0.5em \mathrm{myscle}\kern0.5em \mathrm{in}\kern0.5em \mathrm{lesion}\kern0.5em \mathrm{site}}{\mathrm{Total}\kern0.5em \mathrm{area}\kern0.5em \mathrm{of}\kern0.5em \mathrm{the}\kern0.5em \mathrm{lesion}}\kern0.5em \times \kern0.5em 100 $$

The mean of this ratio was finally recorded for each patient and also for each group. The first image was taken from the surface of the lesion, the second image from the mid part of the lesion, and the third image from the deepest part of the lesion.

### Electromyography

After sonography and determination of the exact location of the gap, needle EMG was carried out by a single blinded physiologist. A 38-mm-long standard concentric EMG needle (38 mm × 0.45 mm; Umbo, Malaysia) was used. Standard filter settings (5 Hz–5 kHz), gain (200 μV/div, changed as needed), and sweep speed (10 ms/div) were used in the EMG system (Natus, Denmark). Subjects lay on their left side, with hips and knees flexed. Their right thighs were grounded electrically. The needle was inserted into the subcutaneous part of the external anal sphincter muscle to a depth of a few millimeters under the mucosa, about 1 cm from the anal orifice. For deeper parts of the external anal sphincter, needle insertions into the muscle were made at the anal orifice, at an angle of about 30° to the anal canal axis. Four quadrants of the external anal sphincter including the gap area marked by sonography were evaluated for spontaneous activity and voluntary motor unit action potentials (MUAPs). For evaluation of MUAPs, patients were asked to squeeze their sphincter, whilst for evaluation of spontaneous activity, patients had to relax the sphincter.

### Statistical analysis

Sample size was determined based on evaluating the difference between recovery percentage in the control group and the hADSC treatment group. Based on the data of previous studies, the rate of recovery from fistula was reported to be 71% following hADSC transplantation and 12% in the control group [23]. Therefore, considering a 95% confidence interval (CI) (α = 0.05) and 80% power (β = 0.2), 11 patients in each group was enough for comparison.

Data were entered into SPSS 21.0 statistical software. Quantitative data were reported as mean and standard deviation (SD) and qualitative data were described as frequency and percentage. Because the data distribution was not normal, nonparametric analyses were used. To assess the difference between qualitative factors in the two groups, Fisher’s exact test was used. To compare age and the percentage of muscle in the injury site after intervention, the Mann–Whitney *U* test was applied. Because the Wexner score was different between the groups before intervention, to compare them after treatment an ANCOVA test (adjusted for the score before intervention) was used. In all analyses, *p* < 0.05 was considered significant.

## Results

### Characteristics of hADSCs

**Fig. 2:**
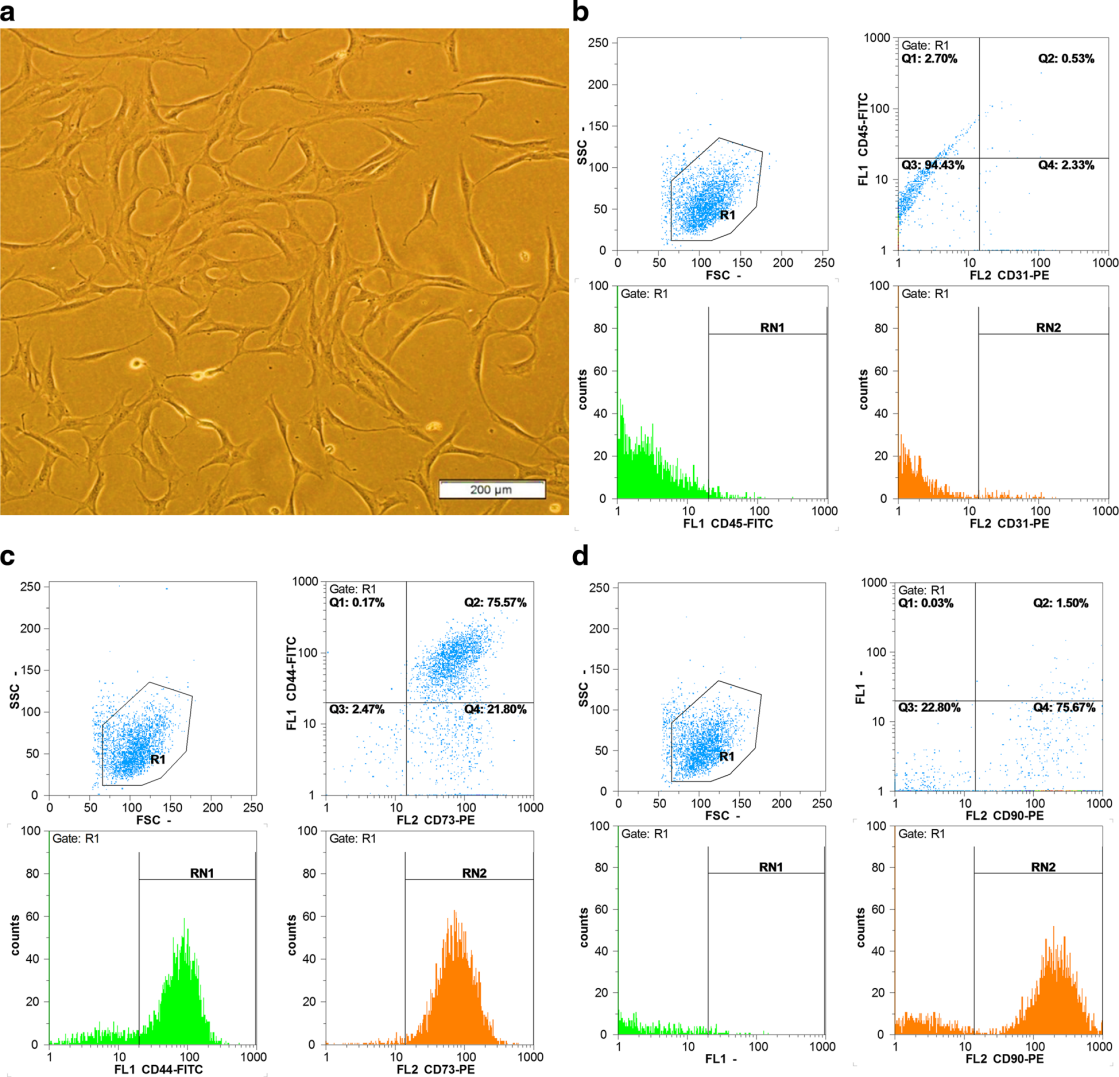
Characteristics of hADSCs. **a** Morphology at the fifth passage (spindle-shaped cells with fibroblast-like appendages are evident). **b**–**d** Flow cytometry analysis of hADSCs (cells express CD73, CD44, and CD90, but not CD31 and CD45). *FSC* forward scatter, *SSC* side scatter

Regarding cell culture, hADSCs were cultured in DMEM/F12 medium with FBS 10%. After the fifth passage, the cells were assessed using an inverted microscope equipped with a camera, which indicated spindle-shaped cells with fibroblast-like appendages (Fig. 2a).

In flow cytometry analysis, hADSCs had special surface markers of mesenchymal cells (positive for CD73, CD44, and CD90). At the fifth passage, a high percentage of cells expressed these markers as CD44 (75.77%), CD73 (97.40%), and CD90 (77.23%). Unlike these markers, hADSCs had no other markers including CD31 or CD45 (negative for these two markers). In fact, cells at the fifth passage expressed a very small percentage of CD31 (2.90%) and CD45 (3.37%) markers (Fig. 2b–d).

### Baseline characteristics of patients

Adult patients with fecal incontinence were recruited for treatment by hADSCs or by placebo (3 ml PBS) from January 2012 to December 2013. Participants were assessed at the time of randomization (baseline) and followed up at 2 months.

A final number of 18 patients, nine in each group, were thus included. The mean age of patients in the hADSC-treated and control groups was 42.63 ± 18.42 and 37.43 ± 14.75 years, respectively (*p* = 0.45). Fourteen patients (77.78%) were female (*p* = 0.71). The baseline characteristics of the patients are presented in Table 2.

**Table 2:** Baseline characteristics of included patients

Variable	Total	Control group	hADSC-treated group	*p*
Age (median, IQR)	40.2 (25)	38.5 (22)	36.0 (22)	0.45*
Gender (*n*, %)				
Male	4 (22.22)	2 (22.22)	2 (22.22)	0.71^#^
Female	14 (77.78)	7 (77.78)	7 (77.78)	
History of GI diseases (*n*, %)				
No	13 (72.22)	6 (77.78)	6 (66.67)	>0.99^#^
Yes	5 (27.78)	2 (22.22)	3 (33.33)	
Number of deliveries (*n*, %)				
0	7 (41.18)	2 (25.0)	5 (55.56)	0.22^#^
1	7 (41.18)	5 (62.50)	2 (22.22)	
2	2 (11.78)	0 (0.0)	2 (22.22)	
3	1 (5.88)	1 (12.50)	0 (0.0)	
Rectal urgency (*n*, %)				
No	18 (100.0)	9 (100.0)	9 (100.0)	>0.99^#^
Yes	0 (0.0)	0 (0.0)	0 (0.0)	
History of anal surgery (*n*, %)				
No	11 (61.11)	5 (55.56)	6 (66.67)	>0.99^#^
Yes	7 (38.89)	4 (44.44)	3 (33.33)	
History of urinary urgency (*n*, %)				
No	18 (100.0)	9 (100.0)	9 (100.0)	>0.99^#^
Yes	0 (0.0)	0 (0.0)	0 (0.0)	

*IQR* interquartile range, *GI* gastrointestinal, *control group* sphincteroplasty alone, *hADSC-treated group* human adipose-derived stem cells + sphincteroplasty

*Based on Mann–Whitney *U* test

^#^Based on Fisher’s exact test

### Safety assessment

All of the hADSC-treated patients were evaluated for safety. No case of discontinuations due to side effects was detected. There were no cases of hemorrhage, need for blood transplants, pain, diarrhea, colon inflammation, infection, allergic reactions, recurrence of disease, readmission, and mortality. In addition, other side effects relating to hematologic, chemistry, pancreas and liver enzyme, stool, and urine analyses as well as cardiovascular, respiratory, digestive, neurologic, skin, and systemic (allergic reaction, headache, fever, and malaise) evaluations were absent in the patients. Only one case (11.11%) of erythema was seen in the site of surgery, which was closely followed; therefore it did not lead to any serious problems for the patient and was relieved completely.

### Patients’ outcome

The mean baseline Wexner score of patients in the control and hADSC-treated groups was 6.0 ± 1.18 and 10.33 ± 0.87, respectively (*p* = 0.02). Two months after surgery, the Wexner scores of the control (*p* = 0.01) and hADSC-treated (*p* = 0.01) groups significantly decreased and reached 2.67 ± 0.62 and 6.44 ± 1.08, respectively. Based on nonparametric ANCOVA, the efficacy of conventional therapy and cell therapy was not significantly different (*p* = 0.36) (Table 2).

**Fig. 3:**
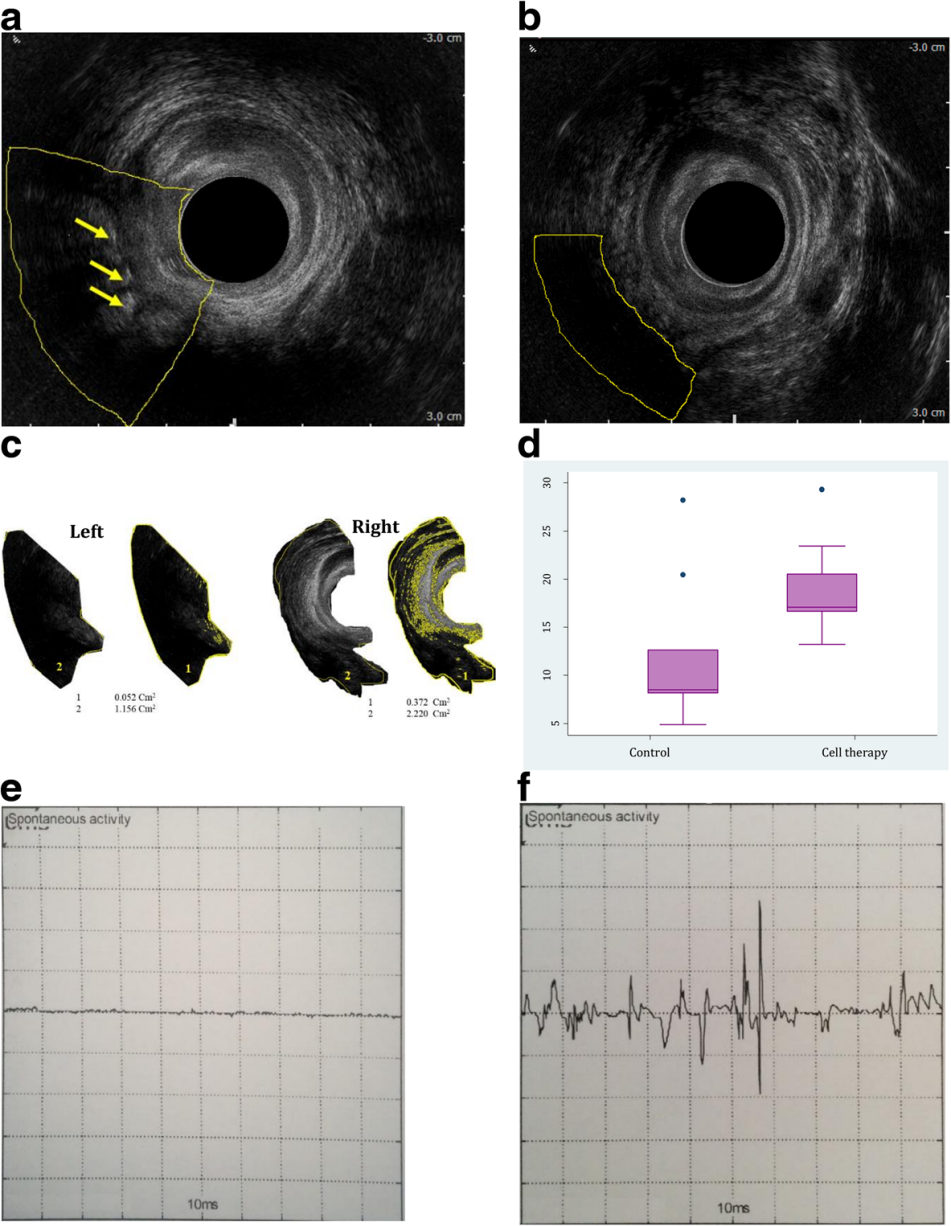
Results of endorectal sonography and EMG 2 months after surgery. **a** Sphincteroplasty alone, fibrous tissue at the gap of the repair site (*arrows*). **b** hADSCs + sphincteroplasty, detecting muscle in the gap created in the repair site. **c** Calculating the ratio of the area occupied by the muscle to total area of the lesion using ImageJ/Fiji 1.46 software (*left*: control group, *right*: cell group). **d** Median percentage of area occupied by the muscle in the control and cell groups (*p* = 0.02). Sample EMG of the control group (**e**) and the cell group (**f**)

The results of endorectal sonography after complete wound healing and at least 8 weeks after surgery and injection of the cells clearly revealed the presence of scattered islands in the repair site compared with the control group (Fig. 3). Measuring the amount of muscle in the repair site using ImageJ/Fiji 1.46 software showed that the mean ratio of the area occupied by muscles was 18.85 ± 5.06% in the cell group and 11.65 ± 7.75% in the control group, which indicates a significant difference (*p* = 0.02) (Table 3).

**Table 3:** Comparison of patients’ outcome between studied groups

Variable	Total	Control group	hADSC-treated group	*p*
Wexner score (mean ± SD)				
Baseline	8.17 ± 3.74	6.0 ± 1.18	10.33 ± 0.87	0.02*
Post intervention	4.56 ± 3.22	2.67 ± 0.62	6.44 ± 1.08	0.36^#^
Standardized mean difference (95% confidence interval)^a^	3.73 (2.61–4.86)	3.63 (2.0–5.07)	3.97 (2.31–5.62)	0.41
Percentage of muscle occupied area (mean ± SD)	15.25 ± 7.34	11.65 ± 7.74	18.85 ± 5.06	0.02*
Electromyography recording (*n*, %)				
Negative	10 (58.82)	9 (100.0)	1 (12.50)	0.002*
Positive	7 (41.18)	0 (0.0)	7 (87.50)	

*Control group* sphincteroplasty alone, *hADSC-treated group* human adipose-derived stem cells + sphincteroplasty, *SD* standard deviation

^a^Based on Hedge’s *g*

*Based on Mann–Whitney *U* test

^#^Based on nonparametric ANCOVA test

EMG in the injected cell group showed that five out of nine views had normal action potentials. One of the cases had no action potential that was consistent with sonography results indicating the lack of muscular islands in the lesion site after injection of cells. In another case, the results of EMG were not recorded due to omission of the test. In one patient, the sphincter had been cut at two sites and a normal action potential was revealed in one site, but not in the other. In another patient, a normal action potential was subsequently recorded at one site and both neurogenic and spontaneous activities were recorded at the other. EMG recordings were negative in all control patients (Table 3 and Fig. 3). Fisher’s exact test revealed that EMG activity was significantly higher in hADSC-treated compared with control patients (*p* = 0.002). No adverse event was seen at the 2-month follow-up.

## Discussion

Simple repair of anal sphincters without further intervention can achieve an acceptable short-term improvement in Wexner scores; however, recurrence of fecal incontinence may be significant after 2 years, with fibrous tissue being insufficiently functional when compared with muscle tissue. Therefore, replacing fibrous tissue with muscle tissue may lead to improved and less deteriorating results in the long term, which was the main hypothesis of the present study. Similarity of the Wexner scores in the cell and control groups might simply be due to the presence of a temporary mechanical barrier and its effect on the Wexner score as a short-term benefit only, at about 2 months following intervention. The true potential benefits of stem cell injection for Wexner score improvement might thus be better judged in the long term, and hence all patients are to be reevaluated after 2 years.

In our study, the site of lesion after simple repair without any other intervention was seen as a uniform black view in ultrasonography, indicating the presence of fibrous tissue. But in the injected cell group, non-homogeneous white islands were visible at irregular intervals, not previously identified in early endo-anal sonography. White islands on the ultrasound view seem to be muscle tissue.

In our assessment, the ratio of the area occupied by the muscle to total area of the lesion showed a 7.91% increase in the cell group compared with the control group. The ability of hADSCs to differentiate into muscle cells has been clearly shown in both in-vitro [25–28] and in-vivo [29–33] studies. In addition to hADSC differentiation into muscle cells, another factor that affects muscle tissue replacement on fibrous tissue is the anti-fibrotic properties of MSCs. As shown by Xiong et al. in 2014 [38], hADSC injection into the submandibular gland in rats reduced fibrous tissue in the gland after stimulation with radiation. Yu et al. suggested in 2010 [39] that hepatocyte growth factor (HGF), originating from MSCs, had anti-fibrotic effects in rats’ livers. hADSCs are the main source for the secretion of growth factors and different types of cytokines such as HGF [40]. HGF is effective in peripheral wound healing through regulating the expression of transforming growth factor beta (TGF-β) and can decrease expression of isoforms of TGF-β, also inhibiting the secretion of TGF-β1 and TGF-β2 [41]. Among the numerous precursors of fibrous tissue, TGF-β1 was the most effective for fibrous tissue formation [42].

The present study was a randomized double-blind clinical trial with small sample size, and therefore generalizability of the results is uncertain. Since hADSC transplantation led to improved EMG findings and showed no undesirable effect, it is suggested to carry out phase 3 and 4 clinical trials to be able to decide on the generalizability of the findings. The short follow-up period is among other limitations of the present study. However, long-term follow-up is ongoing and not yet finished. In addition, the results of the endorectal sonography were not confirmed or substantiated by biopsy or magnetic resonance imaging, which are much better at detecting and differentiating anal structures as well as actual muscle from other types of mucosal tissues. The difference in the speed of closure and wound healing between the cell group and the control group was not assessed in the present study because there was no similar literature on the assessment of the treatment progress of hADSCs after transplantation in repair sites and also because we did not have previous experience of such study. In addition, surgical techniques (such as the size of incisions in the skin for drainage) were not matched between the groups. However, it was qualitatively evident that the duration of the closure and wound healing in the cell group was much faster than in the control group.

Patients evaluated in the current study were heterogeneous and were affected with external anal sphincter damage due to trauma or high trans-sphincter fistula. This heterogeneity might have affected the findings. Therefore, it is suggested to consider the patients’ homogeneity in future clinical trials.

## Conclusions

The results of the current study show that injection of hADSCs in fecal incontinence repair surgery may cause replacement of fibrous tissue with muscle tissue, which exhibits contractile function. This is a key point in treatment of fecal incontinence, especially in the long term, and can be a major step forward in the treatment of such conditions.

However, the present study is only a preliminary report and evaluated heterogeneous patients. Therefore, it is suggested to study the effect of hADSCs on fecal incontinence in future clinical trials with a larger sample size and on a homogeneous group of patients affected with external anal sphincter damage.

## Abbreviations

CI: Confidence interval
EMG: Electromyography
hADSC: Human adipose-derived stromal/stem cell
HGF: Hepatocyte growth factor
MUAP: Motor unit action potential
PBS: Phosphate buffer saline
TGF-β: Transforming growth factor beta
